# Transplantation of a 3D-printed tracheal graft combined with iPS cell-derived MSCs and chondrocytes

**DOI:** 10.1038/s41598-020-61405-4

**Published:** 2020-03-09

**Authors:** In Gul Kim, Su A. Park, Shin-Hyae Lee, Ji Suk Choi, Hana Cho, Sang Jin Lee, Yoo-Wook Kwon, Seong Keun Kwon

**Affiliations:** 1 Department of Otorhinolaryngology-Head and Neck Surgery, Seoul National University Hospital, Seoul, 03080 Republic of Korea; 2 Department of Nature-Inspired Nanoconvergence Systems, Korea Institute of Machinery and Materials, Daejeon, 34103 Republic of Korea; 3 Biomedical Research Institute Seoul National University Hospital, Seoul, 03080 Republic of Korea

**Keywords:** Experimental models of disease, Experimental models of disease, Biomedical engineering, Biomedical engineering

## Abstract

For successful tracheal reconstruction, tissue-engineered artificial trachea should meet several requirements, such as biocompatible constructs comparable to natural trachea, coverage with ciliated respiratory mucosa, and adequate cartilage remodeling to support a cylindrical structure. Here, we designed an artificial trachea with mechanical properties similar to the native trachea that can enhance the regeneration of tracheal mucosa and cartilage through the optimal combination of a two-layered tubular scaffold and human induced pluripotent stem cell (iPSC)-derived cells. The framework of the artificial trachea was fabricated with electrospun polycaprolactone (PCL) nanofibers (inner) and 3D-printed PCL microfibers (outer). Also, human bronchial epithelial cells (hBECs), iPSC-derived mesenchymal stem cells (iPSC-MSCs), and iPSC-derived chondrocytes (iPSC-Chds) were used to maximize the regeneration of tracheal mucosa and cartilage *in vivo*. After 2 days of cultivation using a bioreactor system, tissue-engineered artificial tracheas were transplanted into a segmental trachea defect (1.5-cm length) rabbit model. Endoscopy did not reveal granulation ingrowth into tracheal lumen. Alcian blue staining clearly showed the formation of ciliated columnar epithelium in iPSC-MSC groups. In addition, micro-CT analysis showed that iPSC-Chd groups were effective in forming neocartilage at defect sites. Therefore, this study describes a promising approach for long-term functional reconstruction of a segmental tracheal defect.

## Introduction

The trachea is a rigid fibroelastic organ composed of C-shaped hyaline cartilage, ciliated columnar epithelium, and connective tissue^1^. The tracheal cartilage supports its cylindrical shape and prevents the airway from collapsing during expiration^2^. Tracheal epithelium also plays a critical role in host defense by moving various particles, such as dust, mold, and bacteria^3^. Tracheal disorders commonly arise from intrinsic disorders including infections, inflammatory disorders, trauma, and malignancy^4,5^. These disorders can cause structural problems, such as narrowing and stenosis of the upper airway. In most cases, primary anastomosis is performed after tracheotomy, but if anastomosis is impossible, long reconstruction graft is inevitable. Numerous approaches to regenerate these major structures have been studied^6–8^. However, few studies have successfully undertaken tracheal reconstruction in tracheal defects longer than ~5 in clinical trials, and incomplete regeneration of tracheal cartilage and mucosa can be the main cause of tracheal restenosis^9,10^. To prohibit restenosis, rapid remodeling of the mucosa layer on the inner surface of artificial trachea may be a critical strategy^11^. To this end, we have previously reported enhanced mucosal regeneration using a laminin-coated asymmetrical polymer membrane^12^. However, despite the advantages of bioactive scaffolds, mucosal regeneration on the internal surface of the scaffold cannot maintain the patent airway without tracheal cartilage regeneration. Several studies have demonstrated the effects of tracheal tissue engineering, including approaches based on bioreactor systems and stem cell therapy^13,14^. To our knowledge, few researchers have successfully induced tracheal cartilage into segmental tracheal defects. Cartilage tissue commonly has a low capacity for spontaneous healing owing to its avascularity and low cellularity^15,16^. From this perspective, cell-based therapy has been recognized as a potentially effective approach to healing tracheal cartilage. Chondrocytes have limited proliferation capacity and rapidly lose their differentiated phenotype in culture^17^. Therefore, chondrogenic cells derived from induced pluripotent stem cells (iPSCs) have emerged as a promising source to enhance cartilage regeneration^18–20^. The most significant benefit of iPSC-derived progenitor cells for cartilage regeneration is that they may provide an unlimited supply of chondrogenic cells for *in vivo* applications. In addition, iPSCs can avoid the ethical controversy associated with using human embryonic stem cells (hESCs) in clinic practice.

**Figure 1:**
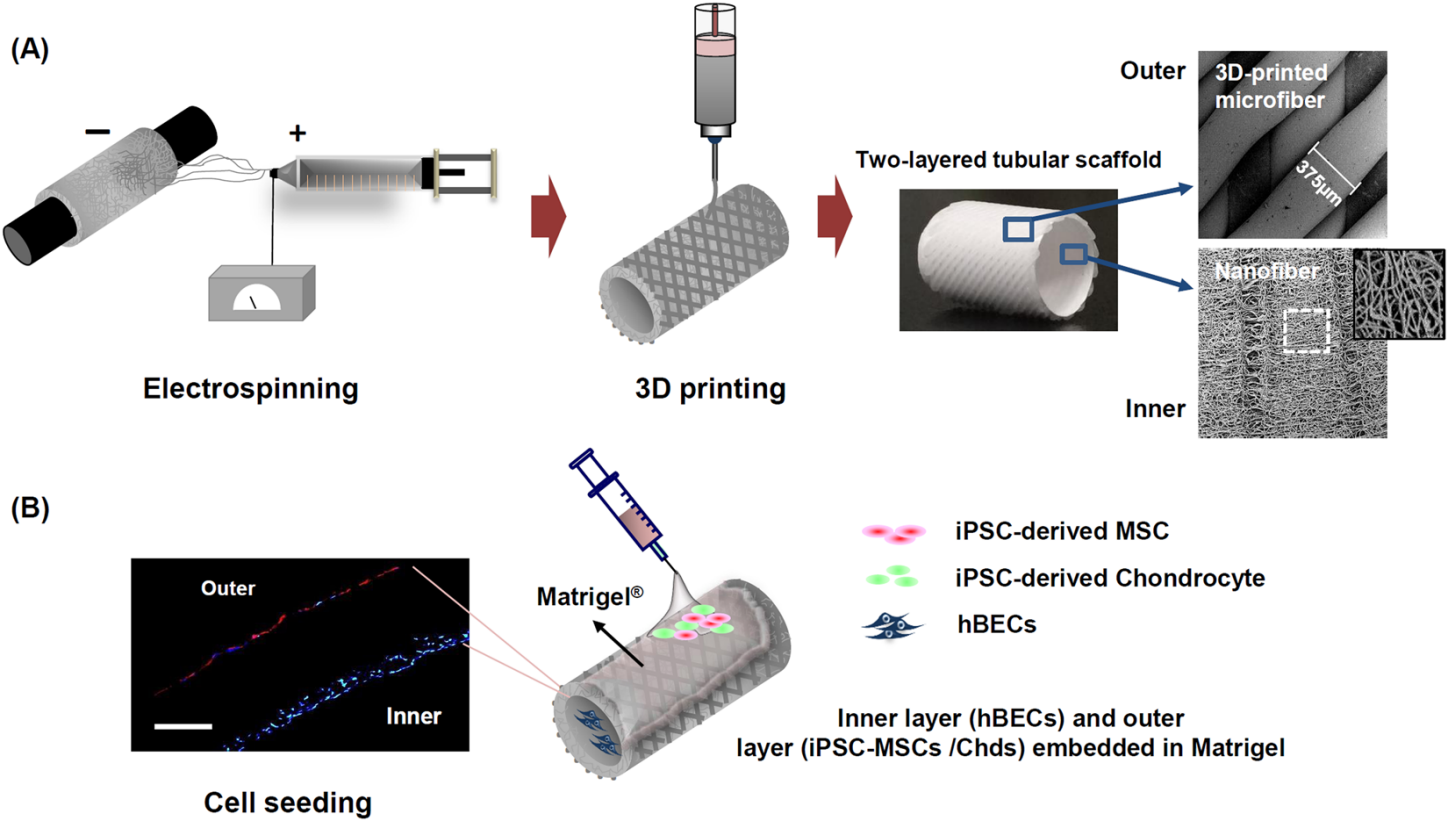
Schematic illustration of the process used to fabricate three-dimensional (3D) tubular artificial tracheal scaffolds and cell seeding thereon. (**A**) Polycaprolactone (PCL) tubular nanofiber layer (inner) was fabricated by an electrospinning technique, and the resulting composite was covered with 3D-printed PCL strands. Surface morphologies of the inner and outer layers of the tubular tracheal scaffold were analyzed by scanning electron microscope (SEM) image. The diameter of the 3D printed strands, which are directly attached to the inner membrane, was measured to be 375 µm on average. (**B**) Induced pluripotent stem cell-derived chondrocytes (iPSC-Chds; encapsulated in Matrigel) and human bronchial epithelia cells (hBECs) were seeded onto the outer and inner layers of the scaffold, respectively. Cells attached to each layer were identified by PKH-26 staining (red color; iPSC-chds) and DAPI (blue color; hBECs) staining (scale bar = 200 μm).

To achieve full regeneration of the tracheal mucosa and cartilage framework, we fabricated a novel two-layered tubular scaffold as an artificial trachea, consisting of electrospun nanofiber (EN; inner) and three-dimensional (3D)-printed microfiber (outer) (Fig. 1A). Further bioactivity for re-epithelialization is conferred to this scaffold through the seeding of human bronchial epithelia cells (hBEC) onto the inner layers of the scaffold. iPSC-derived mesenchymal stem cells (iPSC-MSCs) and iPSC-MSC-derived chondrocytes (iPSC-Chds) were also seeded onto the outer region of the scaffold using Matrigel to promote the regeneration of cartilage tissue (Fig. 1B). We hypothesized that the inner-nanofiber structure can act as a template for the mucosal layer by providing a topographical cues for cell migration; similarly, the 3D-printed microfiber (outer) may provide mechanical strength and flexibility as the framework of the trachea. We therefore evaluated the effect of a new artificial trachea which can enhance the regeneration of mucosal layer and cartilage through analyzing the most optimal combination of iPSC-derived precursor cells, epithelial cells and 3D-printed tubular scaffold in long circumferential tracheal defect (1.5 cm). Furthermore, we investigated whether co-transplantation of iPSC-MSCs and chondrocytes within the artificial trachea led to the enhanced regeneration of tracheal cartilage and tracheal mucosa *in vivo*.

## Results

### Generation of iPSC-MSCs from iPSCs

**Figure 2:**
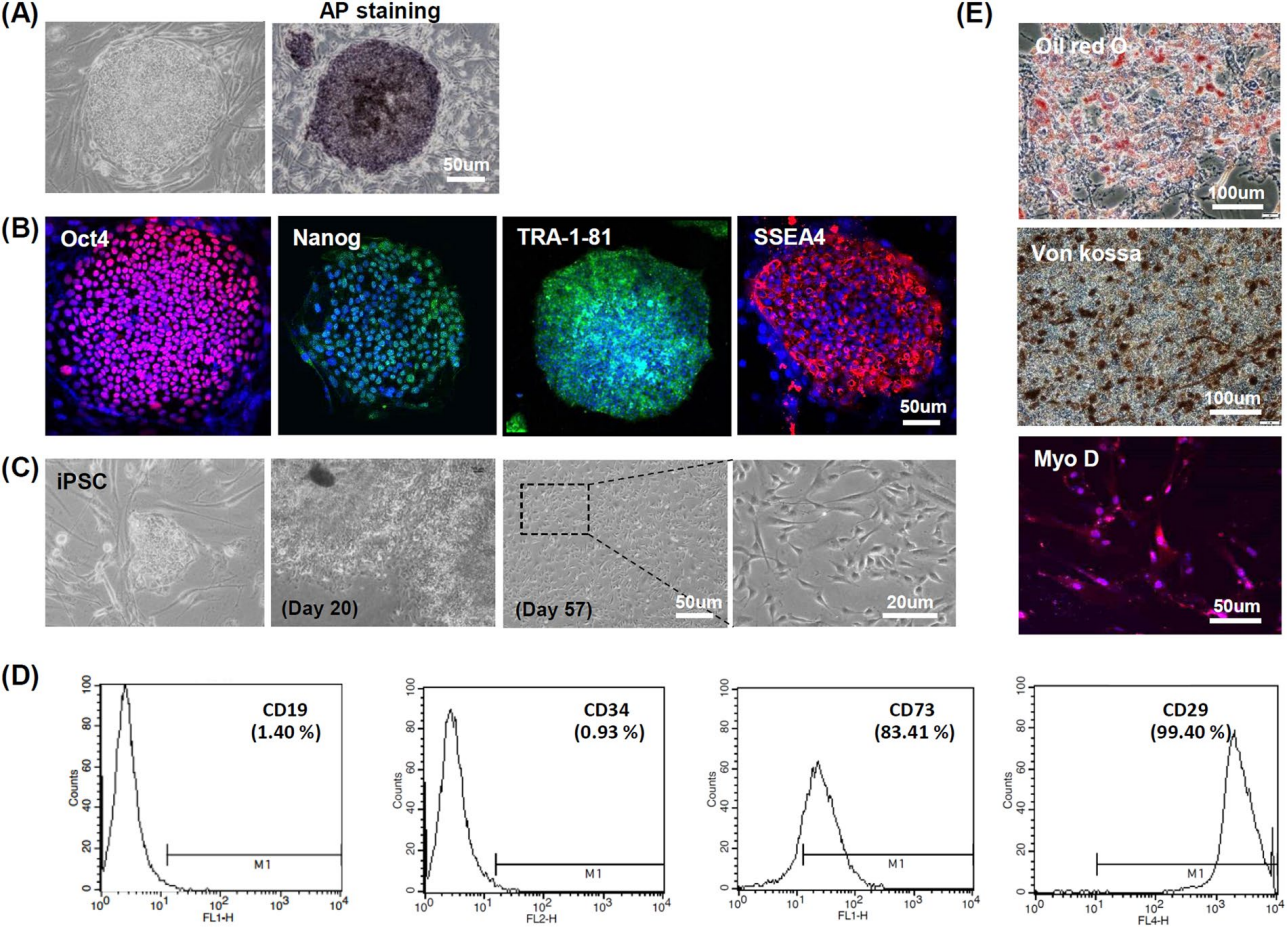
Human iPSCs and basic characterization of iPSC-derived mesenchymal stem cells (iPSC-MSCs). (**A**) Morphology and alkaline phosphatase (AP) activity of iPSC colony. (**B**) Immunostaining of human iPSCs for the pluripotent markers Oct4, Nanog, TRA-1-81 and SSEA4. (**C**) Morphological changes during MSC differentiation via embryonic body cultivation. (**D**) MSC surface marker characterization by FACS analysis. (**E**) Multilineage differentiation of iPSC-derived MSCs. Oil red O, von Kossa, and Myo D (immunostaining) were used to assess the multilineage differentiation of iPSC-derived MSCs for adipogenesis, osteogenesis and myogenesis, respectively.

The morphology of iPSC colonies on the feeder layer was monitored during *in vitro* maintenance via phase contrast microscopy. iPSC colonies were also live-stained with alkaline phosphatase (AP) to confirm early pluripotency. AP-positive iPSC colonies clearly displayed a deep-purple color (Fig. 2A). The pluripotency of the generated hiPSCs was demonstrated by positive immunofluorescence staining for the pluripotency markers OCT4, NANOG, TRA-1-81 and SSEA4 (Fig. 2B). Phase contrast images of Fig. 2C show the morphological changes during MSC differentiation via EB cultivation. After 20 days, most iPSC colonies had lost their morphological features during MSC differentiation. For most colonies, MSC-like cell populations were observed by the 57th day of cultivation. FACS analysis was then performed. iPSC-MSCs were found to be positive for the MSC surface markers CD73 (83.41%) and CD29 (99.40%), and negative for the hematopoietic markers CD19 (1.40%) and CD34 (0.93%) (Fig. 2D). To assess the capability of iPSC-MSCs to differentiate into multiple lineages, Oil red O and von Kossa staining were performed after 21 days of adipogenic and osteogenic differentiation, respectively. As shown in (Fig. 2E), Oil red O staining revealed distinct lipid droplet formation, visualized as a red color; von Kossa staining revealed intense brown/blackish clusters indicating mineralization. In addition, immunofluorescence staining indicated the strong expression of MyoD (red color) at 28 days of myogenic differentiation. To assess teratoma formation, iPSCs (2 × 10^6^) and iPSC-derived MSCs (2 × 10^6^) were implanted into the dorsal subcutaneous tissue of immunodeficient nude mice and evaluated with histological confirmation over a time period of 6 months. H&E stains of iPSC-derived teratomas showed differentiation into all three germ layers (ectoderm, mesoderm, and endoderm), while iPSC-MSCs did not show any evidence for teratoma formation (Supplemental Fig. 1A,B)

### Chondrogenic differentiation of iPSC-derived MSCs

**Figure 3:**
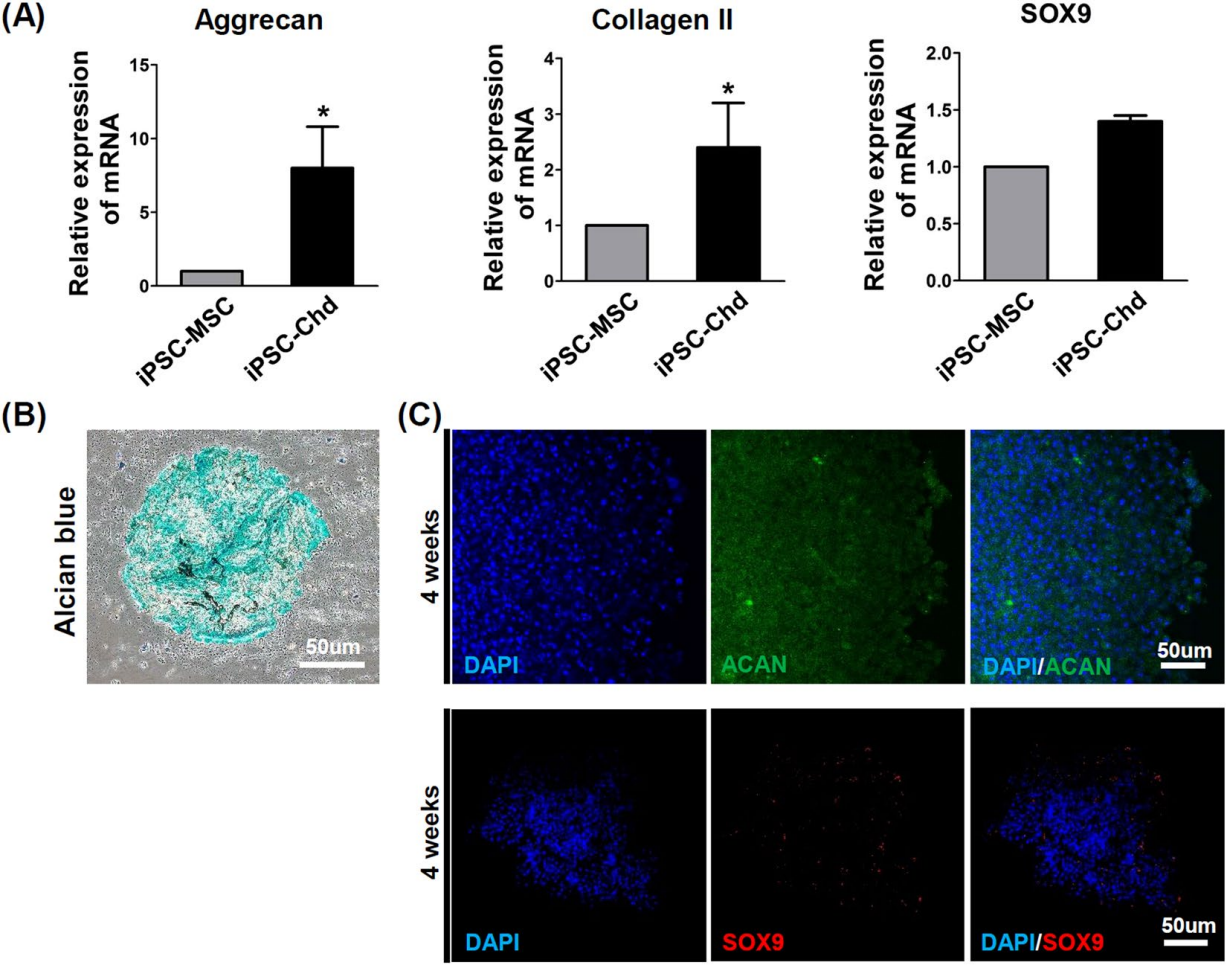
Evaluation of iPS-Chd chondrogenic differentiation *in vitro*. (**A**) RT-PCR for the chondrogenic genes Aggrecan, Collagen II and SOX9 from iPSC-MSCs and iPSC-Chds that underwent chondrogenic induction in pellets. (**B**) Alcian blue-stained images for paraffin-embedded thin sections of iPS-Chd pellets (blue, proteoglycan). (**C**) Immunocytochemistry for chondrogenic markers ACAN and SOX9 from iPS-Chds after 28 days of chondro-induction.

To investigate the chondrogenic differentiation of iPSC-derived MSCs *in vitro*, we induced chondrogenic differentiation in a 3D, high-density pellet culture. The mRNA expression of the chondrogenic markers, namely aggrecan, Col II, and Sox9 are shown in Fig. 3A. Aggrecan and Col II, which are late-stage markers for chodrogenesis, were upregulated in iPSC-derived chondrocytes (iPSC-Chds) throughout the culture period. However, the expression of Sox9, an early marker for chondrogenic differentiation, showed no statistical difference at 28 days. Histological analysis of the cell pellets at day 28 of chondrogenic differentiation revealed positive Alcian blue stains, indicating proteoglycan production (Fig. 3B). Moreover, the immunocytochemistry images also showed abundant expression of late-stage chondrogenic marker, ACAN in the iPSC-chondrogenic pellets at four weeks (Fig. 3C). The Sox9, early marker for chondrogenic differentiation was slightly expressed in the late stage of chondrogenic differentiation (four weeks). Collectively, these results suggest that it may be possible to obtain enriched chondrogenic populations from iPSCs.

### Cell proliferation on two-layered artificial trachea

The nanofibers prepared by electrospinning had an average diameter of 6.94 ± 0.21 µm and mean thickness of 218 ± 28 µm. The average diameter of the 3D-printed microfibers was 375 ± 41, and the strand interval was 286 ± 24 µm (Table 1). SEM images of the 3D printed-tubular scaffold are shown in right column of Fig. 1A. It was observed that the tube wall had an asymmetric column-shaped fiber structure. The inner surface of the tube had ENs, which can effectively guide mucosal tissue induction and improve cell migration, while the outer surface had micro-size fibers (~1000 mm), which can provide mechanical strength and flexibility. The diameter of microfibers (outer) was uniformly distributed, as observed at low magnification (mean diameter, 375 um). Microfiber interstices can also anchor Matrigel encapsulated with iPSC-MSCs. To enhance cell growth on the trachea scaffold, we used a 3D dynamic culture system. A custom-made bioreactor was fabricated according to the dimensions appropriate for rabbit trachea transplantation. The bioreactor was composed of a rotating chamber (modified from a 5.0 mL Eppendorf tube) half-filled with culture medium (Supplemental Fig. 2A). To examine cell proliferation at different rotation speeds in the bioreactor system, we cultured HBECs and iPSC-MSCs on the inner and outer surfaces of the trachea scaffold, respectively. After 5 h of cell adhesion, rotational shear stress was applied to the cells under varying rotation speeds (4, 10, 20, and 30 rpm) and then the cells were monitored for 2 days. Cell proliferation was evaluated using a cell counting method with DAPI (HBECs) and PKH-26 fluorescent staining. As shown in (Supplemental Fig. 2B,C), iPSC-MSCs (red color) on the outer layer grew faster at 20 and 30 rpm compared with 10 rpm. HBECs on the inner layer exhibited no statistically significant difference in growth depending on rotation speed. We therefore opted to use 20 rpm for *in vivo* transplantation.

**Table 1:** Characteristics of the two-layered tubular scaffolds.

	Diameter of ENs (μm)	Thickness of ENs (μm)	Diameter of 3D-PMs (μm)	Interval of 3D-PMs (μm)
Average (μm)	6.94 ± 0.21	212 ± 28	375 ± 41	286 ± 24

[ENs, electrospun nanofibers; 3D-PMs, 3D-printed microfibers].

We performed *in vitro* cell tracking studies to determine how well target cells adhere to the inner and outer surfaces of a scaffold before tracheal transplantation. (Supplemental Fig. 3) shows representative fluorescence images used for cell tracking. On the cryosection slides, we observed PKH-64-labelled iPSC-MSCs (green color) around PCL microfibers (outer region) after 2 days of bioreactor cultivation; PKH-24-labelled HBECs (red color) were detected on PCL nanofibers (inner region). iPSC-Chds were also seeded on the outer layer of the scaffold for *in vivo* studies (data not shown).

To verify the immunomodulatory properties of MSCs *in vitro*, a mixed lymphocyte reaction (MLR) was performed according to the presence or absence of MSCs with peripheral blood mononuclear cells (PBMCs) extracted from healthy donors (Supplemental Fig. 4). As a result, T-cell proliferation was significantly inhibited after 3 days in the MSC group compared with the control group, demonstrating that MSCs can inhibit immune cell proliferation by secreting cytokines.

### Transplantation and endoscopic analysis

**Figure 4:**
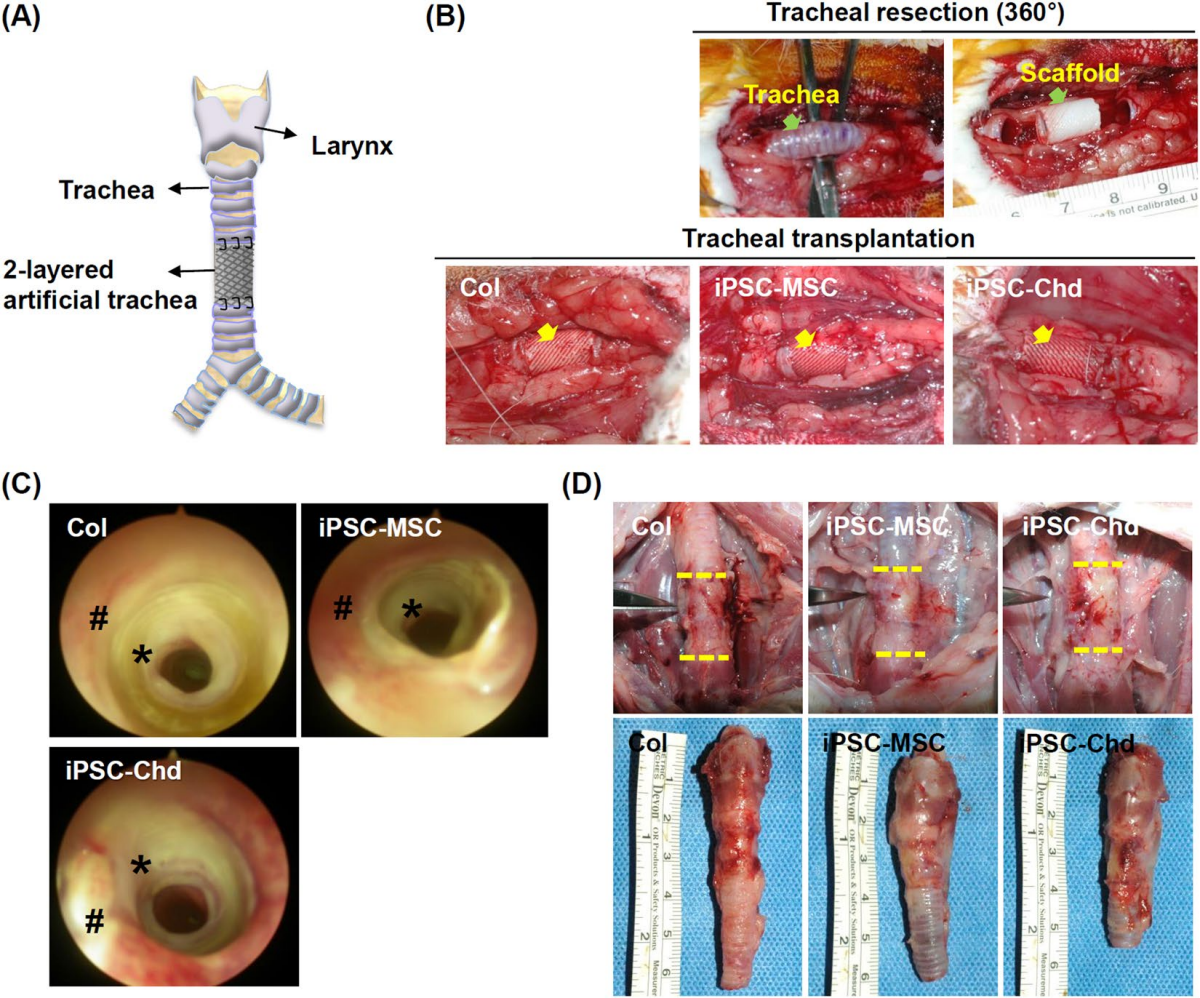
Tracheal transplantation and endoscopic analysis. (**A**) Schematic diagram showing implantation of two-layered tracheal scaffolds in 360° circumferential defects of rabbit trachea. (**B**) End-to-end implantation of artificial tracheal scaffolds (1.5 cm length) in segmental tracheal defects (yellow arrow, implanted scaffold). (**C**) Endoscopic images at 4 weeks. Airway patency was well-maintained in all three groups (#, native trachea; *, implanted tracheal graft). (**D**) Gross findings of implantation site at 4 weeks post-operatively (yellow line, bilateral anastomosis). Macroscopic view of the harvesting of tracheal tissue.

A segmental tracheal defect model was used to examine the effect of 3D printed-artificial trachea on tracheal reconstruction (Fig. 4A). Three hundred and sixty degree circumferential defects (1.5 cm in length) were created on the rabbit trachea, and each scaffold was anastomosed to the edges of the cartilage with absorbable thread (4-0 polydioxanone) (Fig. 4B). The general condition of the rabbits in all groups was good for the 4-week duration of the trial. After 4 weeks, endoscopic examination showed that the artificial materials were covered by epithelium with slight granulation, and there was no evidence of infection or distal accumulation of secretions (Fig. 4C). Airway patency was well-maintained in all three groups without severe granulation. Based on gross appearance, the implant sites were also stable without any dehiscence or collapse at the suture site (Fig. 4D). To investigate the mechanical properties of the artificial trachea before and after tracheal transplantation, we assessed the tensile and flexural stress values of scaffolds harvested at 4 weeks after transplantation and non-treated scaffolds (Supplemental Fig. 5). It was also compared with the mechanical properties of the native trachea. Although flexural stress was slightly reduced in scaffolds implanted for 4 weeks *in vivo*, tensile strength was completely maintained *in vivo* circumstance. In addition, 3D printed-artificial trachea showed similar flexural stress compared to native trachea, and maximal tensile strength was excellent.

### Cartilage regeneration in a rabbit segmental tracheal defect

**Figure 5:**
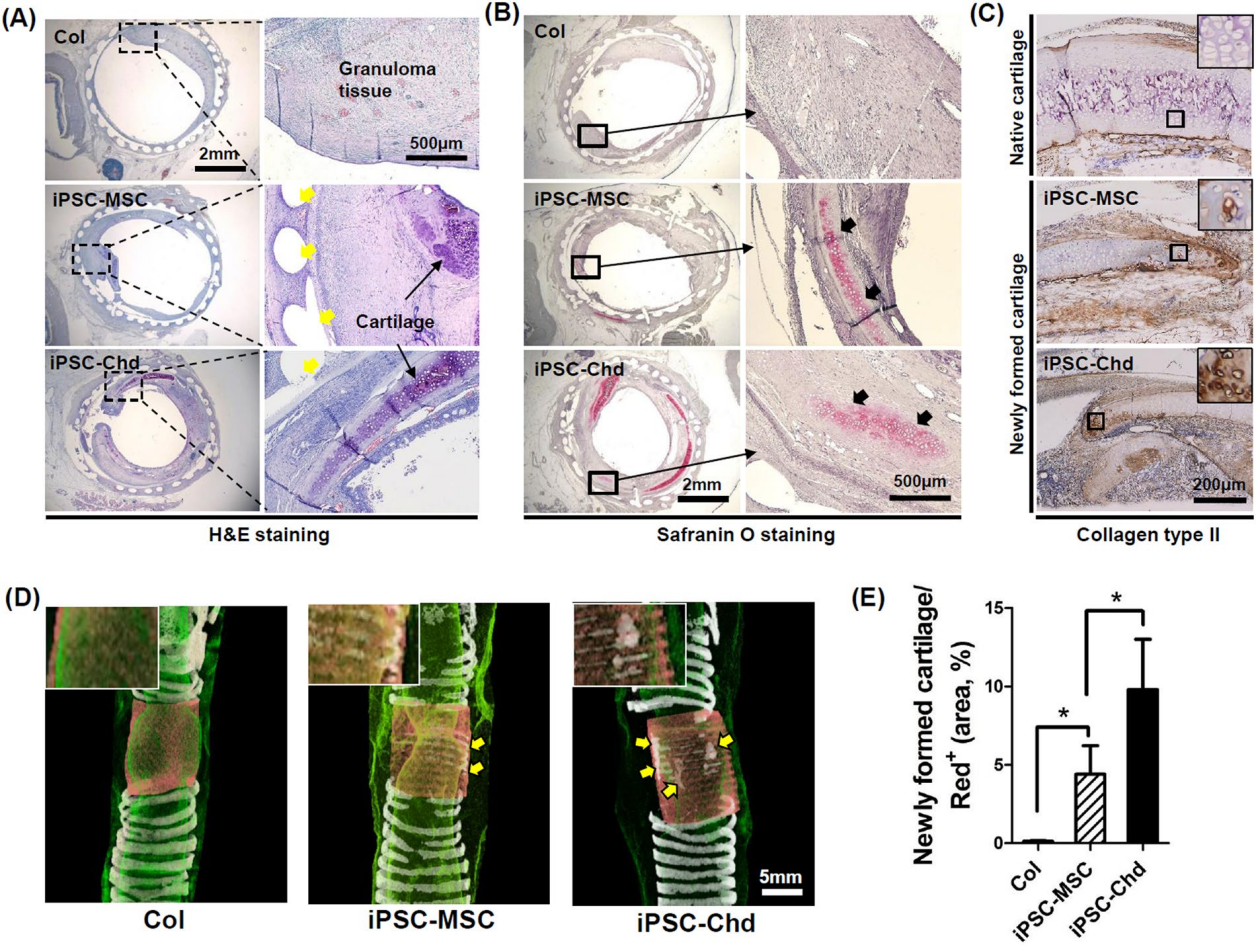
Histologic and radiographic evaluation of tracheal cartilage regeneration. (**A**) H&E staining throughout the entire defect site reveals granuloma tissue formation, location of scaffold (yellow arrows), and newly formed cartilage. (**B**) Safranin O staining throughout the proximal implant site shows the distinct appearance of newly regenerated cartilage (black arrows). Specifically, (**C**) Dab-staining of the area for type II collagen, visualized as a brown color, indicates type II collagen expression in both the iPSC-MSC and iPSC-Chd groups. (**D**) Representative micro-CT images of each group. Native cartilage in trachea is indicated by white rings at both sides of the defect (3D views). Yellow arrows denote newly formed cartilage at defect sites. (**E**) Regenerative tracheal cartilage was calculated based on the quantity of white signal within the area of the region of interest (red^+^) in micro-CT images. (*P < 0.05).

We used H&E staining to assess the histology of transplanted scaffolds across the defect area. The 3D-printed artificial trachea (indicated by yellow arrows) was clearly visible in the tracheal defect site, and slight inflammatory signs or adverse tissue reactions were observed (Fig. 5A). Compared with the acellular scaffold (Col) group, both the iPSC-MSC and iPSC-Chd groups showed far better cartilaginous tissue formation at high magnification, while thick-granuloma tissue formation was observed in the acelluar scaffold (Col) groups (Fig. 5A). In addition, safranin O staining revealed newly formed cartilage in the vicinity of the implants (Fig. 5B). Cartilage formation was much more strongly detected in the iPSC-MSC and iPSC-Chd groups (black arrows) compared with the Col group. More specifically, Dab-positive stains in brown color indicated the biosynthesis of Col II across distal region of newly formed cartilage. (Fig. 5C). We examined iPSC-MSC and iPSC-Chd survival *in vivo* using immunofluorescence staining of human nuclei (Supplemental Fig. 6). The human iPSC-MSCs and human iPSC-Chds showed strong green fluorescence around the 3D-printed PCL (white star), suggesting that the transplanted cells remained stable at the transplantation site. However, a human nuclear antibody and cell surface markers of cartilage did not show co-localization (data not shown). This suggests that human iPSC-MSCs and human iPSC-Chds do not differentiate into and replace the tracheal cartilage. The cells might have had a paracrine effect that facilitated regeneration of host tissues in the grafts. Furthermore, given that iPSC-MSCs modulate the immune response, the presence of immune cells in the graft sites was examined by H&E staining (Supplemental Fig. 7A). We found a significant decrease in mononuclear cells in the iPSC-MSC and iPSC-Chd groups compared with the control groups (Supplemental Fig. 7B). These findings suggest that MSCs committed to the chondrocyte lineage (iPSC-Chd group) still exhibit immunomodulatory effects. Further analysis of transplants via micro-CT revealed cartilaginous regeneration at implant sites (Fig. 5D). The red and white colors in micro-CT images represent implanted scaffold and cartilage, respectively. Both iPSC-MSC and iPSC-Chd groups showed distinct cartilage masses (marked in yellow arrow) in tracheal defects, but the Col group showed few signs of cartilaginous matrix formation. The newly formed cartilage spread well along the 3D-printed mesh structure, especially in the iPSC-Chd group. Most notably, quantitative analysis using Image J confirmed that the percentage of newly formed cartilage was significantly higher in the iPSC-Chd groups than the iPSC-MSC groups (Fig. 5E). These results suggest that the pre-seeding of iPSC-chondrocytes on the scaffold plays an important role in facilitating cartilage formation.

### Mucosal regeneration in a rabbit segmental tracheal defect

**Figure 6:**
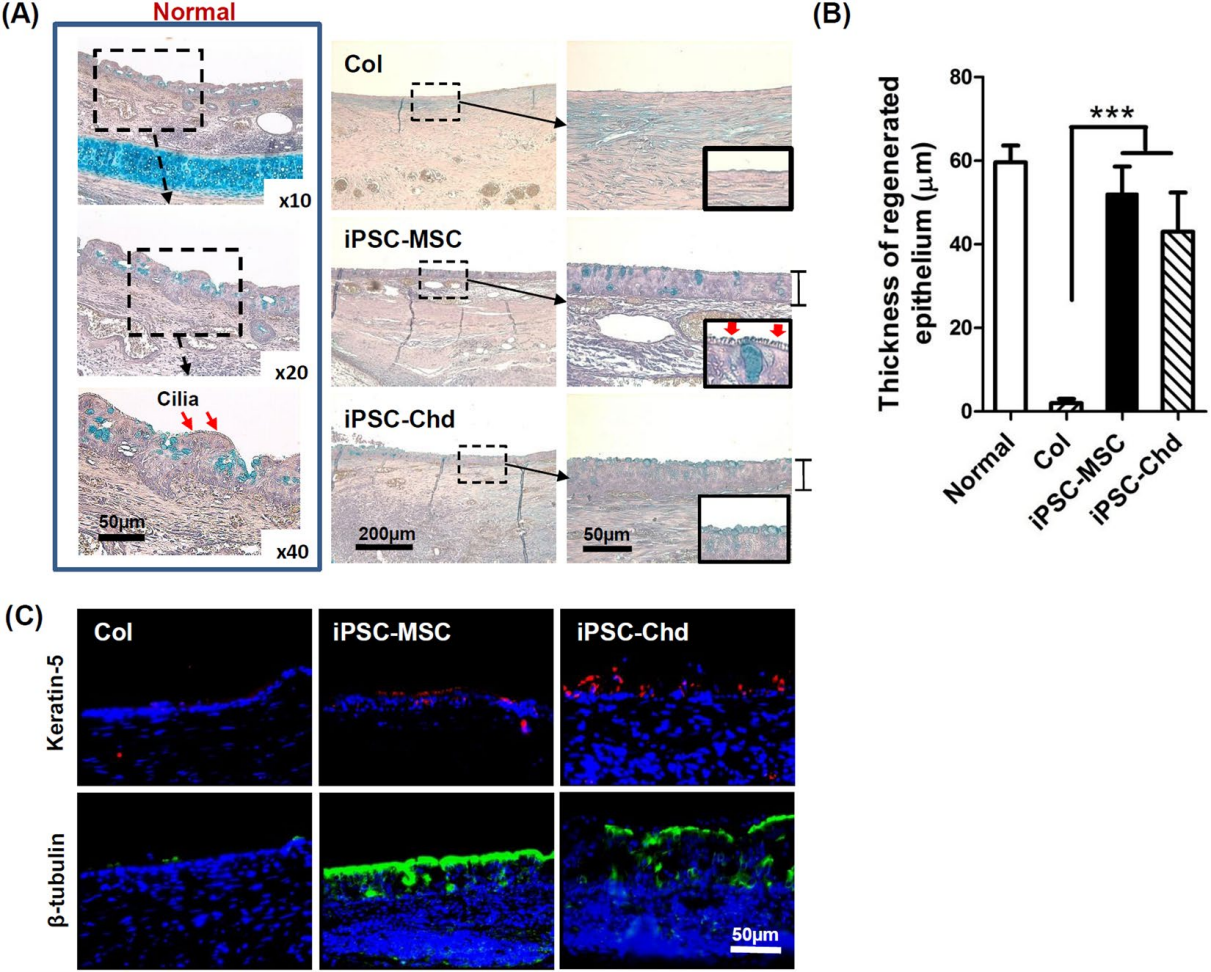
Histological and immunohistochemical evaluation of tracheal mucosal regeneration. (**A**) Alcian blue staining revealed mucus production (blue color) through the regenerative tracheal epithelium at 4 weeks after implantation. In particular, cilia were clearly revealed in the iPSC-MSC group (red arrow). (**B**) Quantitative analysis of the thickness of regenerative epithelium 4 weeks after surgery. Experimental groups seeded with iPSC-MSCs and iPSC-Chd had a significantly greater epithelial thickness than the Col groups (***P < 0.001). (**C**) Immunofluorescence staining for keratin-5 and β-tubulin in implant sites. Keratin-5 (red), and β-tubulin (green) are markers for basal cells and cilia, respectively. The iPSC-MSC and iPSC-Chd groups showed positive signals for keratin-5 and β-tubulin in the regenerative mucosal layer.

Although no epithelium was observed on the surface in the Col group, regenerative epithelium was observed on tracheal internal lumen in both the iPSC-MSC and iPSC-Chd groups (Fig. 6A). Notably, high-magnification images showed that the Col and iPSC-Chd groups had no cilia on the internal surfaces. In the iPSC-MSC groups, we could identify cilia on top of the regenerative epithelium. In particular, the epithelial thickness for both the iPSC-MSC and iPSC-Chd groups was significantly greater than that of Col groups (Fig. 6B). Additionally, immunofluorescent staining for Keratin5 and β-tubulin was carried out to identify mucosal cells from the newly reconstructed tracheal epithelium (Fig. 6C). Keratin-5 expression (a basal cell marker) was observed through the regenerative mucosal layer in the iPSC-MSC and iPSC-Chd groups. In particular, expression of β-tubulin (a marker of cilia) was predominant in the iPSC-MSC group compared with the iPSC-Chd group. iPSC-MSCs clearly showed a critical effect in promoting mucosal regeneration. However, the human nuclear antibody and cell surface markers of the epithelium showed no co-localization (data not shown), suggesting a paracrine effect of the iPSC-MSCs.

### Co-transplantation of chondrocytes and iPSC-MSCs for enhancing cartilage regeneration

**Figure 7:**
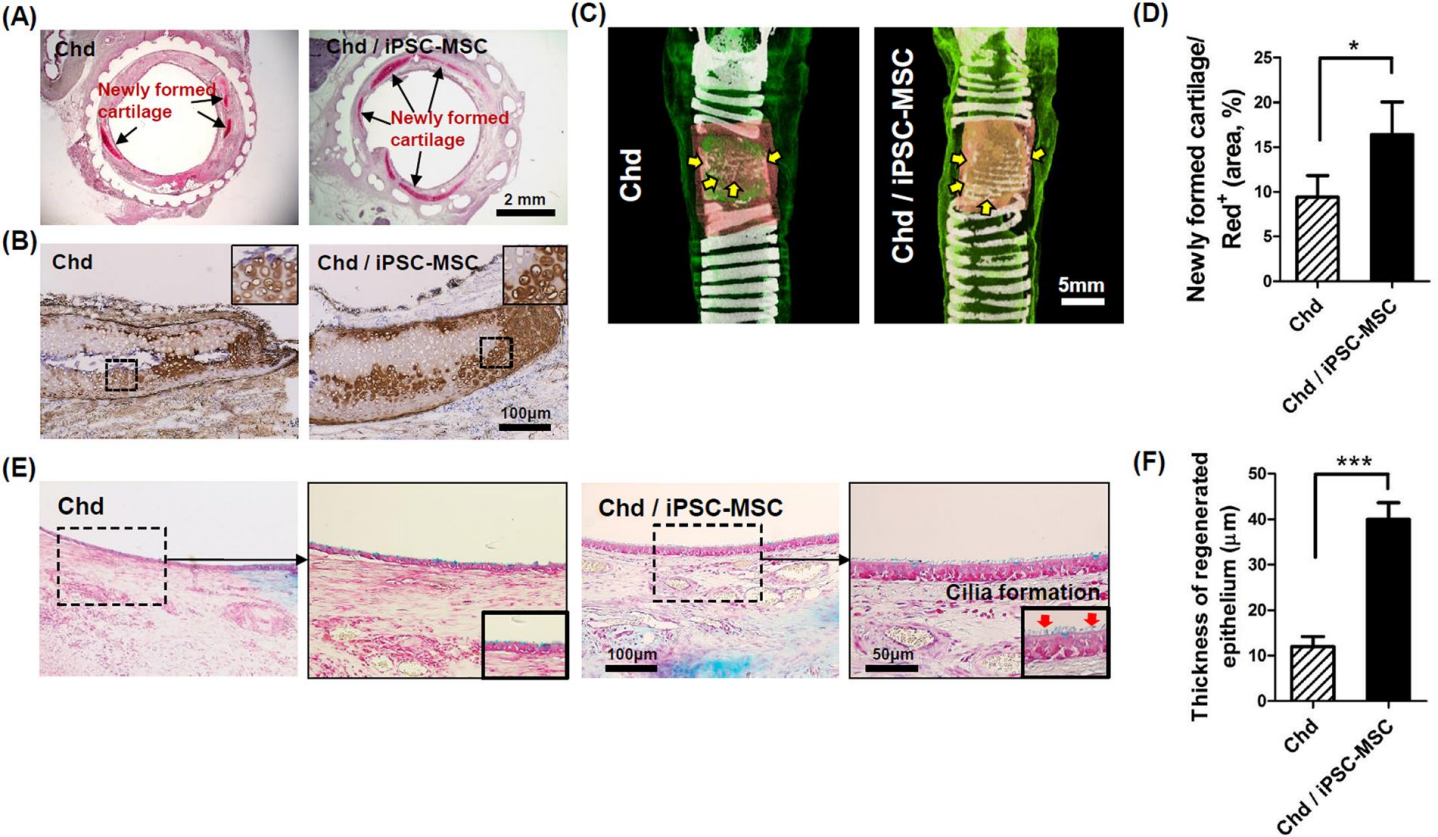
Tracheal reconstruction through co-transplantation of primary chondrocytes and iPSC-MSCs. (**A**) Newly formed cartilage (arrow, red color) is observed as assessed by safranin O staining. (**B**) Type II collagen-specific immune-dab staining shows a brown positive signal with a lacunae structure. (**C**) The defect site was also analyzed at week 4 for cartilage regeneration via reconstruction of micro-CT images. Yellow arrows indicate regenerative cartilage in the region of the tracheal scaffold implant. (**D**) Regenerative tracheal cartilage was calculated based on the quantity of white signal within the area of the region of interest (red^+^) in micro-CT images. (*P < 0.05). (**E**) Alcian blue staining shows re-epithelialization on the luminal surface in segmental tracheal defects. In particular, the Chd/iPSC-MSC group exhibited thick epithelial regeneration and cilia formation. (**F**) Quantitative analysis of the thickness of regenerative epithelium 4 weeks after surgery. The epithelial thicknesses were significantly increased in the Chd / iPSC-MSC groups than in the Chd groups (***P < 0.001).

To enhance the regeneration of tracheal cartilage and mucosa simultaneously, we attempted to transplant 3D-printed artificial tracheas with chondrocytes co-cultured with iPSC-MSCs (Supplemental Fig. 8). Safranin O staining and Col II immunostaining were performed to assess cartilage regeneration in the Chd and Chd/iPSC-MSC groups. Safranin-O staining clearly showed the regeneration of rich cartilaginous ECMs (red color) around the periphery of a 3D printed artificial trachea (Fig. 7A). DAB-positive stains (brown color) revealed the presence of Col II, which is the most abundant protein during cartilage remodeling, around the two-layered tubular scaffold (Fig. 7B). A representative micro-CT image of 3D-reconstructed cartilage is also shown in Fig. 7C. As expected, extensive cartilage regeneration was observed in both groups. Quantitative analysis showed that newly formed cartilage was significantly increased in the Chd/iPSC-MSC groups than in the Chd groups (Fig. 7D). Most notably, the cartilage regenerative capacity of the Chd/iPSC-MSC group increased dramatically compared to the iPSC-MSC and iPSC-Chd groups. (Supplemental Fig. 9). Re-epithelialization was also assessed via Alcian blue staining (Fig. 7E). While the Chd/iPSC-MSC groups showed a thick epithelium layer with cilia, the Chd group displayed a thin epithelium layer without cilia (Fig. 7F). In particular, the epithelial thickness for all iPSC-MSC-seeded groups was significantly greater than that of other experimental groups (Supplemental Fig. 10). Collectively, these results suggest that our two-layered artificial trachea co-cultured with Chds and iPSC-MSCs exerted a strong positive effect on *in vivo* tracheal cartilage regeneration. Furthermore, we demonstrated that iPSC-MSCs facilitate epithelialization of hBEC in our two-layered tracheal scaffold.

## Discussion

Successful tracheal reconstruction using tissue-engineered artificial trachea requires biocompatible 3D constructs comparable with natural trachea, coverage with ciliated respiratory mucosa, and adequate cartilage remodeling^21–24^. Many studies have documented the induction of tracheal mucosa and cartilage using a variety of scaffolds. For instance, Park *et al*. developed a 3D-printed tracheal graft with mechanical properties similar to those of native trachea to regenerate tracheal cartilage^25^. They also reported that the combination of 3D-printed PCL scaffolds and human turbinate mesenchymal stromal cell (hTMSC) sheets enhanced tracheal epithelialization in non-circumferential tracheal defects in a rabbit model^26^. However, their results are very limited, since they did not use segmental tracheal defects for 3D tracheal reconstruction. At present, despite advances in tissue engineering techniques, it is still difficult to apply fully tissue-engineered tracheal replacements for segmental tracheal defects (>5 cm). This is partly because implant sites typically experience air flow, neck movement, and bacterial infection during the remodeling process. Thus, clinical trials of artificial trachea have been largely unsuccessful^27,28^. New strategies for tracheal regeneration are therefore required.

In the present study, we assessed the feasibility of a 3D-printed tracheal graft seeded with various cells for regenerating segmental tracheal defects. Among its structural features, the inner nanofiber structure can provide morphological properties similar to extracellular matrix, which may help cell adhesion and migration as well as prevent the penetration of granulation tissue. Nanofiber structures have already been investigated as a promising substrate for re-epithelialization in wound healing applications^29,30^. Therefore, we cultured hBECs on a nanofiber layer for regeneration of tracheal mucosa, and confirmed its bioactivity in a bioreactor system (Supplemental Fig. 1B,C). Furthermore, 3D-printed microfibers were deposited on the tubular nanofiber to provide mechanical strength, flexibility, and porosity (Fig. 1A). The 3D printing technique also enables the production of a defect-specific implant that precisely matches the size of the tracheal defect. The 3D-printed porous structures also utilize a Matrigel coating on the scaffold surface for cell attachment. We previously confirmed that Matrigel-coated scaffolds exhibit greater cell adhesion efficiency compared with naked 3D-printed tubular scaffolds (without Matrigel) in bioreactor culture (data not shown). We speculate that the Matrigel anchor in the interstitial space could increase cell stabilization, and thus therapeutic efficacy. In previous studies, we already demonstrated that 3D-printed tubular structures not only provide mechanical stabilization in vascular grafts but can also successfully integrate with the adjacent trachea in tracheal reconstruction^31,32^.

Currently, the contribution of adult MSCs and MSC-derived chondrocytes to cartilage regeneration is well understood^16,20,33^. Adult MSCs have been tested for their clinical applications in the orthopedic and cardiac fields^34^. However, the isolation of MSCs necessitates painful and invasive procedures. In addition, the advanced age of donors and limited culture time hinder the therapeutic application of MSCs and the potential to differentiate harvested cells into functional tissues. The use of iPSCs may circumvent these problems. iPSCs offer several advantages for cartilage regeneration over adult MSCs and chondrocytes^35^. Most adult MSCs and chondrocytes exhibit rapid decrease of proliferation and differentiation potential with increasing time in culture^36^, while undifferentiated hiPSCs can be expanded indefinitely.

In the present study, iPSCs were successfully differentiated into MSCs with high efficiency via intermediate embryoid body formation. The MSC phenotypes were also characterized by flow cytometry and immunofluorescence (Fig. 2D,E). Chondro-induced pellets demonstrated robust production of chondrogenic markers, and strongly expressed Aggrecan and Collagen II (Fig. 3A). Recently, several studies have investigated the therapeutic effects of iPSC-Chds on cartilage repair in osteochondral defect models. Nejadnik *et al*. reported that the overall histological score of repaired cartilage was significantly higher for human iPSC-Chds compared with human iPSC-derived MSC implants in arthritic joints^37^. Similarly, Ko *et al*. demonstrated that osteochondral defects implanted with human iPSC-Chds resulted in a significantly higher quality of cartilage repair compared with the control (bone marrow MSC-derived chondrocytes)^17^.

Newly formed cartilage was identified around the 3D-printed tubular scaffold through safranin O staining and Col II immuno-staining after 4 weeks of implantation. Interestingly, micro-CT 3D analysis also indicated the formation of new cartilage across the central region of the implant site. We do not expect that iPSC-Chds were directly transdifferentiated into cartilaginous tissue. However, iPSC-Chds detected with anti-human nuclei antibodies were observed around PCL microfibers (Supplemental Fig. 6). These data do not address the mechanisms underlying the differentiation of iPSC-Chds into cartilage, and it remains unclear whether grafting iPSC-Chds should regenerate tracheal cartilage beneath the PCL microfibers. We speculate that cytokines, growth factors, and extracellular matrix components released from iPSC-Chds may affect chondrogenic remodeling. Despite their potential advantages, the use of hiPSC-Chds for cartilage regeneration is limited by the inefficiency and complexity of the chondrogenic differentiation processes.

Especially, a more abundant production of cartilaginous ECM was prominently observed around the anastomotic site by Col II immune-staining (Fig. 5C). These results suggest that cartilage regeneration could be maximized by cartilaginous microenvironments and various cytokines around native cartilage. Zhao and his colleagues have demonstrated a mechanism by which conditioned media harvested from cultured auricular chondrocytes promote chondrogenic differentiation of bone marrow-derived MSCs^38^. Therefore, we hypothesized that application of primary chondrocyte may be more effective for cartilage regeneration. In addition, we attempted the co-administration of rabbit chondrocytes and iPSC-MSC for the simultaneous regeneration of cartilage and mucosal layer. Furthermore, we investigated synergistic effects on tracheal cartilage regeneration through co-transplantation of primary chondrocytes and iPSC-MSCs. Quantification of new cartilage formation indicated that the Chd/iPSC-MSC groups formed significantly more neocartilage compared with the iPSC-Chd group (Supplemental Fig. 7). From these results, we can infer that the iPSC-MSCs increase the viability of chondrocytes available for cartilage remodeling, thereby promoting chondrogenic differentiation. Particularly, the iPSC-MSCs have shown sufficient efficacy for tracheal mucosal regeneration (Supplemental Fig. 8).

Several reports have demonstrated that epithelial regeneration is enhanced by various cells and bioactive molecules, such as growth factors and cytokines^13,27^. Each of these factors has distinct effects on re-epithelialization. The use of MSCs is considered a superior therapeutic option for epithelial remodeling because of the greater differentiation potential of MSCs^39^. Studies have shown that MSCs can induce angiogenesis, resulting in improved regeneration of the tracheal mucosa^12^. Furthermore, recent studies demonstrated that MSCs can enhance angiogenesis via integrin receptors^40^. The paracrine effects of MSCs lead to regulation of the tissue microenvironment and ultimately to restoration of damaged tissue^41^. Thus, MSCs exhibit strong immunomodulatory properties and regenerative paracrine effects by producing various cytokines, growth factors, and extracellular matrix^42,43^. As shown in Fig. 6A, Alcian blue staining revealed ciliated columnar epithelium only in the iPSC-MSC groups. Ciliated epithelium is a functional feature of mature tracheal epithelium, and its development is critical for successful epithelial regeneration. In addition, immunofluorescence staining revealed stronger expression of β-tubulin, which is associated with cilium formation, in the iPSC-MSC group than iPSC-Chd group (Fig. 6C). These data indicate that iPSC-MSCs play a role in accelerating the re-epithelialization process. However, we did not detect any specific co-localization between human nuclei and mucosal surface markers by immunofluorescence staining (data not shown). We speculate that paracrine mediators of human cells (hBECs and iPSC-MSCs) promote regeneration of host tissues in the grafts. Previously, we reported that MSCs could transdifferentiate into endothelial cells expressing vWF^12^, and that neovascularization provided a favorable environment by supplying oxygen and nutrition to transplanted grafts^39,44^. Although there was no validation of neovascularization, it was assumed that the angiogenic properties of MSCs provided appropriate microenvironments for enhancing re-epithelialization. Furthermore, MSCs protect human cells through the induction of immune tolerance (Supplemental Fig. 7), and enable long-term survival *in vivo* from immune heterogeneity. Specifically, the hMSCs have been reported to alter cytokine secretion profiles of dendritic cells (DCs), naïve and effector T cells (T helper cells) and natural killer cells to induce more anti-inflammatory or tolerant phenotypes^45^. Therefore, iPSC-derived MSC protected iPSC-Chds and it made long-term survival of iPSC-Chds possible. Collectively, these results suggest the clinical potential of human iPSCs in trachea regeneration.

## Conclusions

In this paper, we have described an innovative method for tracheal reconstruction in a rabbit model of a segmental tracheal defect. Our findings are the first to show that a 3D-printed nanofiber artificial trachea combined with various cell types (iPSC-derived MSCs and chondrocytes; hBECs; autologous chondrocytes) can regenerate into a functional, cartilaginous, epithelialized airway. Future studies using large animal subjects are required to further understand the role of cartilage and mucosal regeneration in segmental tracheal reconstruction.

## Materials and Methods

### Fabrication and characterization of the two-layered tubular scaffold

#### Generation of tubular nanofibers via electrospinning

Polycaprolactone (PCL) tubular ENs were prepared according to the following procedure. Briefly, PCL (MW 80 kDa) was dissolved in 1,1,1,3,3,3-hexafluoro-2-propanol (HFIP; Sigma-Aldrich, MO, USA) solvent to produce a 10% (by weight) solution. For electrospinning, the prepared PCL solution was drawn into a syringe with a blunt metal needle (22 G, Kovax-needle, Korea Vaccine Co., Ltd., Korea) and electrospun on rotating stainless-steel mandrels (diameter = 8 mm) at a distance of 30 cm between the needle tip and rotating collector. The power supply (Nano NC, Korea) was set to a high-voltage direct current of 23 kV potential. The feeding rate of the solution was fixed at 1 mL/h using a syringe pump. A tubular nanofiber layer was fabricated from the surface of the mandrel rotating at 3.14 m/s. The resultant PCL nanofiber was dried in a vacuum oven (40 °C) overnight to eliminate the residual solvent.

#### Manufacture of tubular tracheal scaffold by 3D printing

3D-printed PCL microfibers were produced using a rapid prototyping system^31^. Briefly, PCL pellets (MW 45 kDa) were melted at 100 °C in a heating cylinder, and 3D-printed strands were extruded using a custom-made 3D printer (3D bioprinting system, Korea Institute of Machinery & Materials, Korea). The 3D printing equipment consists of a dispenser, nozzle, compression/heat controller, 4-axes machine with a mandrel axis, and software system. 3D-printed PCL microfibers were plotted in a layer-by-layer manner along the rotation of the mandrel axis through the extrusion of melted PCL onto the tubular PCL nanofiber under an air pressure of 300 kPa. The nozzle size and distance between strands were 300 and 1200 μm, respectively. The 5-mm mandrel axis holding the PCL nanofiber was rotated between layers to produce a patterned 0°/45° porous structure. The surface morphology of the 3D-printed tubular scaffold was visualized by scanning electron microscopy (SEM; Model S-3000N; Hitachi, Japan) (Fig. 1A; right column).

### iPSC culture

#### Culture of iPSCs

Human iPSCs were derived from human skin cells using the Yamanaka’s method previously described^46^. A mitomycin C (Sigma-Aldrich, MO, USA)-treated Sandos Inbred Mice (SIM) embryo-derived thioguanine- and ouabain-resistant (STO) feeder layer, in a 0.1% gelatin coated tissue culture plate, was used for the maintenance of iPSCs. iPSCs were maintained in human iPSC culture media DMEM/F12 (Thermo Fisher, USA), 20% knockout serum replacement (Thermo Fisher, USA), 2 mM L-glutamine (Thermo Fisher, USA), 1% non-essential amino acids (Thermo Fisher, USA), 0.1 mM 2-mercaptoethanol (Thermo Fisher, USA), 50 units penicillin and 50 mg/mLstreptomycin (Thermo Fisher, USA), and 10 ng/mLbFGF.

#### Generation of iPSC-derived MSCs and *in-vitro* differentiation to several lineage

An embryonic body (EB)-based method for deriving MSCs from iPSCs was carried out in multiple steps. (1) iPSC colonies were detached after treatment with 0.5 unit/mL Dispase (Gibco, Grand Island, NY) for 30 min. (2) The detached iPSC colonies were incubated in bacterial dishes for 14 days to form EBs. The EB medium, iPSC medium without bFGF, was changed every other day. (3) After the selection of well-rounded EBs (about 250 μm) under the microscope, the EBs were reattached on a 0.1% gelatin-coated dish. The medium, which was composed of DMEM low glucose (Gibco, Grand Island, NY), 10% fetal bovine serum (FBS) (Gibco, Grand Island, NY), and 1% antibiotic–antimycotic (Gibco, Grand Island, NY) was changed every 3 days for 16 days. (4) The outgrowing cells from EBs were expanded by transfer to and culture in a 0.1% gelatin-coated dish with microvascular endothelial cell media-2 (EGM-2 MV) medium (Lonza, Basel, Switzerland). The derived cells were expanded by subculture for more than 150 days. For adipogenic differentiation, MSCs were seeded at 2.5 × 10^5^ cells/35 mm^2^ in a 0.1% gelatin-coated culture plate. Differentiation was initiated 3 days later, once cells had reached an overgrown state. The adipogenic differentiation media contained alpha-minimum essential medium (α-MEM; Invitrogen, CA, USA), 10% FBS (Gibco, Grand Island, NY), 1 mM dexamethasone, 0.5 mM isobuthylxanthine (all from Sigma), and 10 μg/mL insulin. The media was changed every 3 days for 21 days. For histological evaluation of differentiated adipocytes, cells were stained using Oil Red O. For osteogenic differentiation, MSCs were seeded at 1 × 10^5^ cells/35 mm^2^ in a 0.1% gelatin-coated culture plate. Differentiation was initiated after cells were attached. The osteogenic differentiation media consisted of α-MEM (Invitrogen, CA, USA), 10% FBS (Gibco, Grand Island, NY), 10 mM β-glycerol phosphate, 0.1 μM dexamethasone, and 20 μM ascorbic acid (all from Sigma, MO, USA). The medium was changed every 3 days for 28 days. Osteogenically differentiated MSCs were confirmed using von Kossa staining. For myogenic differentiation, MSCs were cultured in α-MEM (Invitrogen, CA, USA) supplemented with 20% FBS (Gibco, Grand Island, NY) for 3 weeks. Differentiated myocytes were examined via immunofluorescence using rabbit polyclonal anti-MyoD (1:100; Santa Cruz Biotechnology, CA, USA)^47^. Alexa Fluor 594 goat anti-rabbit IgG (Invitrogen) was employed as secondary antibody.

#### Chondrogenic differentiation of mesenchymal stem cells (MSCs)

iPSC-derived MSCs were cultured in MSC-GM (Lonza, Basel, Switzerland) media in 0.1% gelatin-coated tissue culture plates. For chondrogenic differentiation, cells were detached using 0.25% trypsin-EDTA (Invitrogen, CA, USA) and differentiated under 3D pellet culture conditions. Centrifuged MSCs were resuspended in 500 mL of chondrogenic differentiation media (100X Chondrogenic Supplement added to chondrogenic base media [all from R&D Systems]) in 15-mL tubes. Cells were centrifuged at 200 g for 5 min at room temperature, and the tubes were then placed upright in a 5% CO_2_-supplied 37 °C incubator with the lids slightly open to permit gas exchange. The medium was changed 3 times a week for 28 days. Differentiated chondrocytes were stained using Alcian blue solution.

#### Immunocytochemistry analysis of iPSCs and differentiated chondrocytes

For immunocytochemistry analysis, iPSCs were fixed using 4% paraformaldehyde and blocked with 1% bovine serum albumin in 1X PBS; 0.1% Triton X-100 was added to blocking solution to detect nucleus-localized protein. The primary antibodies, anti-Oct4 (1:100; Santa Cruz Biotechnology, CA, USA), anti-Nanog (1:100; Santa Cruz Biotechnology, CA, USA), anti-TRA-1-81(1:100; Santa Cruz Biotechnology, CA, USA), and anti-stage-specific embryonic antigen 1(SSEA1) (1:100; Santa Cruz Biotechnology, CA, USA) were diluted in antibody diluent solution (Invitrogen, CA, USA) and samples were incubated at 4 °C overnight. Suitable secondary antibodies (donkey anti-rabbit Alexa Fluor 488, goat anti-mouse Alexa Fluor 555, and goat anti-rabbit Alexa Fluor 555; all from Invitrogen, CA, USA) were used, and 4′-6-diamidino-2-phenylindole (DAPI) (1:2000; Thermo Fisher, USA) was used for counterstaining. To prepare chondrocyte samples for immunocytochemistry, chondrocyte pellets were embedded in optimal cutting temperature (OCT) compound (Sakura) after fixation in 4% paraformaldehyde and frozen sections were prepared. Chondrocyte-specific markers were confirmed from chondrocyte frozen sections using anti-Sox9 (1:50; Santa Cruz Biotechnology, CA, USA) and anti-ACAN (diluted to 1:50; Santa Cruz Biotechnology, CA, USA) primary antibodies. Compatible secondary antibodies (donkey anti-rabbit Alexa Fluor 488 and goat anti-rabbit Alexa Fluor 555, 1:100 respectively; Invitrogen, CA, USA) were used, and DAPI (1:1000) was used for nucleus counterstaining. Images were obtained using a confocal microscope (LSM 510 Meta, Carl Zeiss, Oberkochen, Germany).

#### RT-qPCR

Differentiated chondrocytes were harvested 28 days after differentiation was initiated. Total RNA was extracted using TRIzol Reagent (Invitrogen, CA, USA), and 1 μg of RNA was used for reverse transcription with the ReverTraAce qPCR RT Master Mix (Toyobo, Osaka, Japan). Real-time PCR was carried out on the Applied Biosystems 7500 Real Time PCR System. Expression data were normalized to 18 S rRNA and calculated using the 2^(−∆Ct)^ method. Primers are listed in Supplemental Table 1^48^.

#### Teratoma formation (*in vivo*)

To confirm the pluripotency of iPSCs (2 × 10^6^) and iPSC-MSCs (2 × 10^6^) cultivated on the feeder layer, each cell type was injected into the dorsal regions of 6-week-old immunodeficient (NOD/SCID) mice (Orient Bio, Sungnam, Korea). When the resulting teratomas were removed 12 weeks later, the samples were paraffin-embedded, then serially sectioned (5 µm) using a microtome (Leica Microsystems, Germany). The three germ layers were confirmed by hematoxylin and eosin (H&E) staining. Images were analyzed using a light microscope (Olympus BX43, Tokyo, Japan).

#### Cell proliferation efficiency using a bioreactor system

Once 3D-printed tracheal scaffolds were prepared, they were sterilized under ultraviolet light for 1 h, pre-wet with ethanol for 10 min, and washed three times in phosphate-buffered saline (PBS). Primary bronchial epithelial cells (HBECs; PCS-300-010), purchased from ATCC, were used without modification (passage #3). PKH-26-labeled HBECs (1 × 10^6^ cells) were seeded onto the inner surface of each scaffold using infiltration methods with 1 mL bronchial epithelial cell basal medium (CC-3171; Lonza, Basel, Switzerland). To achieve efficient attachment on the outer surface of the scaffold, dissociated hiPSC-derived MSCs were suspended at a density of 1 × 10^6^ cells/mL in Matrigel (354234; Corning) containing endothelial cell basal medium-2 (EBM-2) [CC-3156; Lonza, Basel, Switzerland] supplemented with EGM-2MV SingleQuots [#CC-4147, Lonza, Basel, Switzerland], and the suspension was then applied in an even coating on the outer surfaces of the scaffolds. Cell-seeded-scaffolds were tightly fitted into acrylic holders in culture chambers of the custom-made bioreactor (Supplemental Fig. 1A). The culture chamber was then filled with the EBM-2 medium and rotational shear stress was applied at 37 °C under a 5% CO_2_ humidified atmosphere. Cell proliferation was examined at 2 days under different rotation rates (4, 10, 20, and 30 rpm) using a cell counting method with DAPI (HBECs) and PKH-26 fluorescent staining (PKH-26 red-fluorescent cell linker mini kit; Aldrich-Sigma). Fluorescent images were captured under an Olympus BX43 microscope and then counted in five high-power fields per slide in triplicate by a blinded observer.

#### Mixed lymphocyte reaction assay

PBMCs were isolated from whole peripheral blood obtained from a New Zealand white rabbit (Orient Bio, Korea) by density gradient centrifugation and were cryopreserved until use. The supernatant of iPSC-MSC cultures was collected after 3 days of culture in proliferation medium. hBECs (1 × 10^4^/well) were pretreated with 10 μg/mL mitomycin C (Abcam) for 4 hours before culture. PBMCs (1 × 10^5^/well) were co-cultured with or without iPSC-MSCs in a 24-well plate pre-cultured with hBECs. T-cell proliferation was measured at an absorbance of 570 nm using the alamarBlue assay (alamarBlue™ Cell Viability Reagent; Invitrogen).

### Surgical procedures

A rabbit segmental tracheal defect was created using 4-month-old male New Zealand white rabbits weighing 3.15–3.44 kg. Prior to scaffold implantation, each cell type was seeded onto the inner (PKH26-labelled hBECs; 1 × 10^6^ cells) or outer [PKH64-labelled (Aldrich-Sigma) iPSC-MSCs or iPSC-Chds encapsulated in Matrigel; each 1 × 10^6^ cells] surfaces of the scaffolds and pre-incubated in growth media for 2 days using the bioreactor system (20 rpm). Surgical procedures were subsequently performed under general anesthesia induced by the intramuscular injection of zoletil and xylazine (50 mg/kg and 4.5 mg/kg, respectively). The neck areas were shaved with clippers. After sterile preparation and draping, a midline vertical incision was made, and dissection of the fat and muscular planes was performed down to the trachea. After exposing the trachea, a 1.5-cm circumferential tracheal wall defect was created for scaffold placement^49^. Each tracheal graft was positioned in the defect, and was anastomosed to the outward boundaries of the tracheal wall with 4–0 Vicryl sutures (Ethicon). Experimental animals were randomly divided into three groups (n = 5 per group): 1) acellular scaffold coated with collagen (Col), 2) scaffold seeded with iPSC-MSCs (outer) and hBECs (inner), and 3) scaffold seeded with iPSC-Chds (outer) and hBECs (inner). To enhance the regeneration of tracheal mucosa and cartilage simultaneously, we additionally performed tracheal transplantation of 3D printed-artificial tracheas with chondrocytes co-cultured with iPSC-MSCs. Rabbit chondrocytes (Chds) were isolated from articular cartilage fragments of New Zealand White rabbits (16 weeks old, weighing 3.0–3.4 kg; Orient Bio, Seongnam, Korea). Briefly, the rabbit articular cartilage was removed from the knee joint by sterile dissection, finely minced, and suspended in PBS. The tissue fragments were washed several times and fully digested at 37 °C in 0.25% collagenase (Sigma, MO, USA) in DMEM (Gibco, Grand Island, NY) for 3 h. The released chondrocytes were centrifuged at 1200 rpm for 10 min and washed twice with PBS. Once counted, the cells (1.5 × 10^5^ cells/cm^2^) were plated and cultured in DMEM containing 10% FBS. The chondrocytes were passaged once prior to use. For tracheal transplantation, 1.5-cm segmental tracheal defects were created as described above. Prior to scaffold implantation, each cell type was also seeded onto the inner (hBECs; 1 × 10^6^ cells) or outer (Chds or Chds co-cultured with iPSC-MSCs in Matrigel; each 1 × 10^6^ cells) surfaces of the scaffold and pre-incubated in growth media for 2 days using the bioreactor system (20 rpm). Surgical procedures were all performed as described above. The experimental groups were divided into two groups (n = 4 per group): 1) scaffold seeded with Chds (outer) and hBECs (inner) and 2) scaffold seeded with Chds/iPSC-MSCs (outer) and hBECs (inner). All rabbits were sacrificed at 4 weeks post-operation.

### Mechanical properties

Two mechanical parameters (tensile stress, flexural stress) of 3D printed/NFs scaffolds were measured before and after implantation over a period of 4 weeks using an ultimate tensile test machine (AG-5000G, Shimadzu, Japan) equipped with a 10-kgf load cell at a crosshead displacement speed of 20 mm/min. Trachea of normal rabbit was used as a control group.

### Endoscopic airway examination

All animals were sacrificed at 4 weeks post-operation. Immediately after euthanization, a rigid endoscope (Richards, Knittlingen, Germany) was inserted orally and fixed in position so as to provide the best view of the trachea. Images were taken of each animal’s airway with a digital camera (E4500, Nikon, Japan) connected to the endoscope.

### Micro-CT analysis

The whole rabbit trachea was dissected after macroscopic observation of the reconstructed trachea. The tracheal tissues were fixed with 10% formalin for 3 h and subjected to radiographic analysis using a micro computed tomography (CT) scanner (NFR Polaris-G90; NanoFocusRay, Jeonju, Korea). The scanned CT images of each specimen were taken at settings of 65 kV, 60 µA, and 500 ms, with a resolution of 512 × 512 in Digital Imaging and Communication in Medicine (DICOM) format. 3D images of each rabbit’s trachea were reconstructed using software (Lucion; Infinitt Healthcare, Seoul, Korea). The area of regenerated cartilage was calculated from 3D reconstructed images using Image J software (NIH, USA).

### Histological and immunohistological analysis

Immediately after micro-CT scanning, all specimens were fixed in a 10% neutral formalin solution and embedded in paraffin. Thin sections were cut through the implanted areas in the transverse plane and stained using H&E, safranin O, and Alcian blue staining, following standard histological techniques. Safranin-O staining was carried out using Safranin-O solution (0.1%, w/v) and Fast Green solution (0.001%, w/v) after deparaffinization of specimens with Histoclear II and ethanol. For alcian blue staining, paraffin slides were treated with 3% acetic acid solution for 4 min and stained with alcian blue at 37 ° C for 15 min. The sections were then counterstained with eosin. Histological images were obtained in triplicate from each group using a light microscope (Olympus, Tokyo, Japan). The distribution of lymphocytes across the implantation sites was observed by H&E staining in a high-power field at 400× magnification. The number of lymphocytes per high-power field was determined in five different fields per sample (n = 5 per group). The thickness of the regenerative epithelium was measured using Image J based on the Alcian blue staining. For immunohistochemistry, endogenous peroxidase was inactivated using 3% hydrogen peroxide in methanol for 30 min. The tissue sections were washed with PBS and then exposed to 3% bovine serum albumin (BSA) to block nonspecific reactions. Sections were subsequently incubated with aniti-Keratin5 (1:50 dilution; mouse monoclonal antibody; ABIN126702) and β-tubulin (1:200 dilution; mouse monoclonal antibody; MA5-11732) along with the secondary antibodies: Alexa Fluor 594 goat anti-mouse (ab150116; Abcam, USA) and Alexa Fluor 488 goat anti-mouse (ab150113; Abcam, USA), respectively. Ketatin5 and β-tubulin are the markers of basal and ciliated columnar cells, respectively. Primary antibody (mouse monoclonal anti-human nuclei PMAB1281; EMD Millipore]) and secondary antibody (Alexa Fluor 488 goat anti-mouse [ab150113]) were also administered to track the presence of transplanted human cells. Images were captured using a fluorescence microscope (BX43-32FA1-S09; Olympus Optical, Japan). Five areas (200× magnification) were examined per slide (n = 6 per group) by a blinded observer.

### Statistical analysis

All the data are expressed as mean ± standard deviation. Statistically significant difference is obtained from one-way analysis of variance (ANOVA) with a posthoc, Bonferroni’s multiple comparison tests using GraphPad Prism 5 (GraphPad Software, LaJolla, CA). A significant difference is marked as *(p < 0.05), **(p < 0.01), or ***(p < 0.001).

### Study approval

All procedures involving human subjects were approved by the Seoul National University Hospital Review Board (H-0908-036-290), and written informed consent was obtained from tissue donors. All methods were performed in accordance with relevant guidelines and regulations. All animal experiments were approved by the Animal Care Committee of the Seoul National University Hospital (IACUC No. 15-0268-S1A1), and surgical procedures were performed according to animal care guidelines.

## Supplementary information

Supplementary Information.
