# Supplementary material for: Transplantation of a 3D-printed tracheal graft combined with iPS cell-derived MSCs and chondrocytes
Source: Sci Rep. 2020 Mar 9;10:4326. doi: 10.1038/s41598-020-61405-4 (PMC7062776; doi:10.1038/s41598-020-61405-4)
Supplement: Supplementary file 1 — Supplementary Information. [file 41598_2020_61405_MOESM1_ESM.docx]

**Supplemental Information**

**Transplantation of a 3D-printed tracheal graft combined with iPS cell-derived MSCs and chondrocytes**

*In Gul Kim, Su A Park, Shin-Hyae Lee, Ji Suk Choi, Hana Cho, Sang Jin Lee, Yoo-Wook Kwon^*^, and Seong Keun Kwon^*^*

**Supplementary Table 1**. Primer sequence list.

|  | Primer sequence | Temperature |
| --- | --- | --- |
| Sox9  ([NM_000346.3](https://www.ncbi.nlm.nih.gov/entrez/viewer.fcgi?db=nucleotide&id=182765453)) | (F)GACTTCCGCGACGTGGAC | 60ºC |
|  | (R)GTTGGGCGGCAGGTACTG |  |
| Collagen type II alpha 1  ([NM_001844.4](https://www.ncbi.nlm.nih.gov/entrez/viewer.fcgi?db=nucleotide&id=111118975)) | (F)GGCAATAGCAGGTTCACGTACA | 60ºC |
|  | (R)CGATAACAGTCTTGCCCCACTT |  |
| Aggrecan  ([NM_001135.3](https://www.ncbi.nlm.nih.gov/entrez/viewer.fcgi?db=nucleotide&id=256017258)) | (F)TCGAGGACAGCGAGGCC | 60ºC |
|  | (R)TCGAGGGTGTAGCGTGTAGAGA |  |
| 18S RNA | (F)GGCCCTGTAATTGGAATGAGTC | 60ºC |
|  | (R)CCAAGATCCAACTACGAGCTT |  |


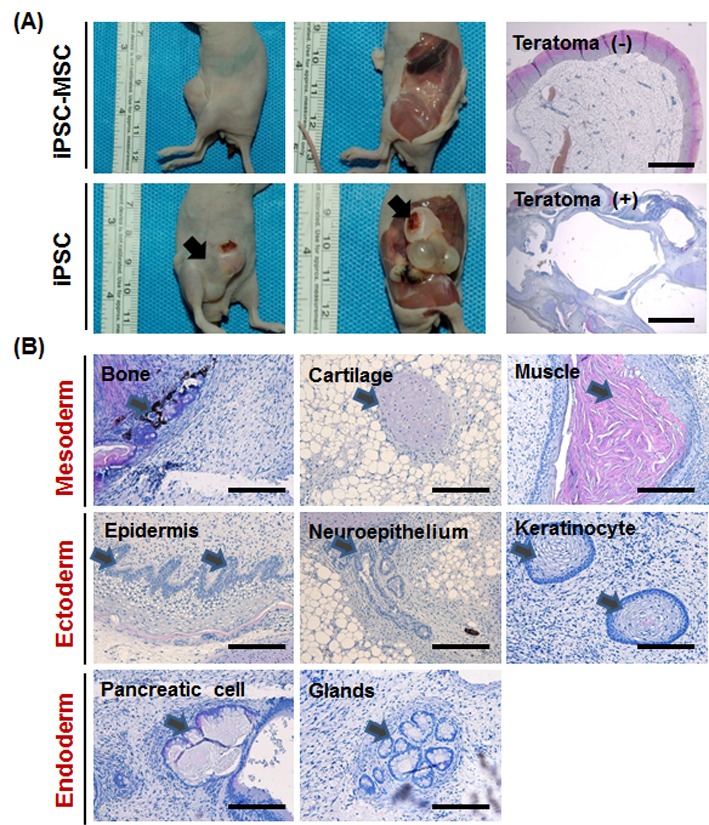


**Figure 1.** Teratoma formation analyses of immunodeficient (NOD/SCID) mice. iPSCs were subcutaneously injected in each mouse. (A) Photograph of NOD/SCID mice 12 weeks after iPSC injection: the teratoma is clearly observed. The arrows indicate the teratoma formation site. The tissue sections were derived from teratomas and stained with hematoxylin and eosin (scale bars = 2 mm). (B) Mesoderm, ectoderm and endoderm layers were clearly observed (scale bars = 100 μm).

**
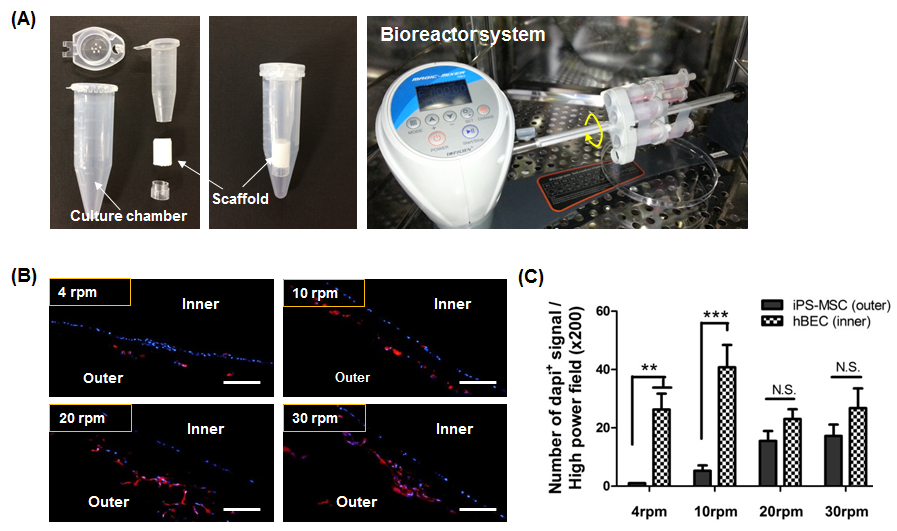
**

**Figure 2.** Dynamic culture of the two-layered tracheal scaffold using custom-made bioreactor device. (A) The culture chamber is composed of two parts that hold the tubular scaffold. Culture chambers inserted into the rotator were subjected to rotational flow. Each cell immersed in medium is exposed to a fluid flow-induced shear stress. (B) Cell proliferation at different rotation speeds (4, 10, 20, 30 rpm) were examined by PKH-26 staining (red color; iPSC-MSCs) and DAPI (blue color; hBECs) staining (scale bar = 200 μm). (C) The growth rate of each cell was quantitatively analyzed by DAPI signal at various rotation speeds (***P* < 0.01 or ****P* < 0.001).


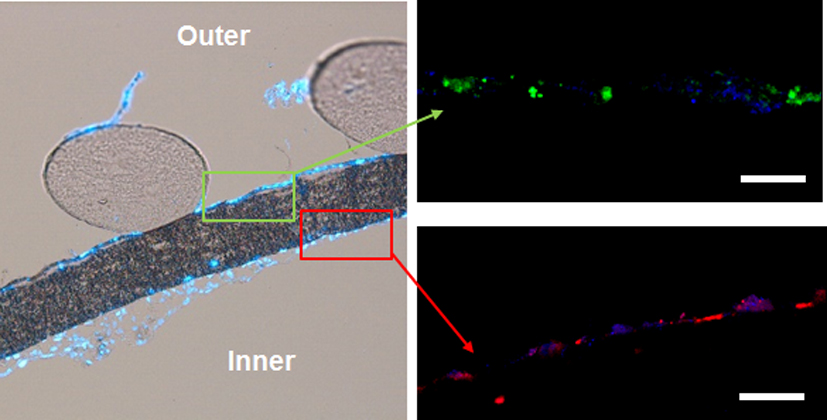


**Figure 3.** Fluorescence tracking of iPSC-MSCs (green) and hBECs (red) cultured on tracheal scaffolds prior to tracheal transplantation (scale bar = 50 μm).


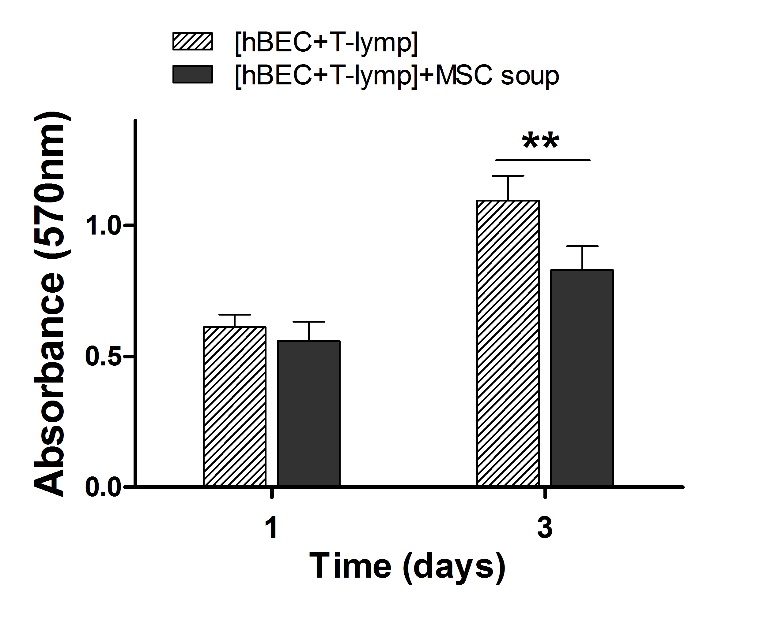


**Figure 4.** MLR assay of rabbit T-lymphocyte on hBECs co-cultured with or withour MSC soup (T-lymp, T-lymphocyte). Cell proliferation was examined on day 1 and 3 via a alamarBlue assay (n=5 each group).


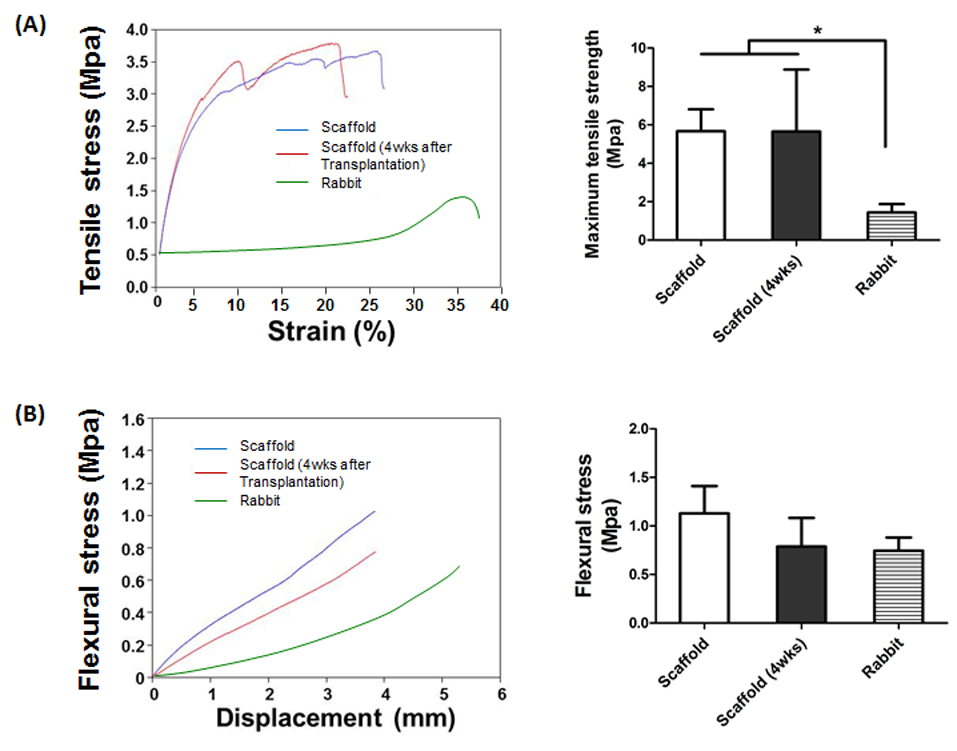


**Figure 5.** Maximum tensile strength (A) and flexural stress (B) of the two-layered tracheal scaffold before and after transplantation. Both scaffold groups had significantly higher tensile strength than rabbit trachea, but there was no statistically significant difference in flexural stress (**P* < 0.05).


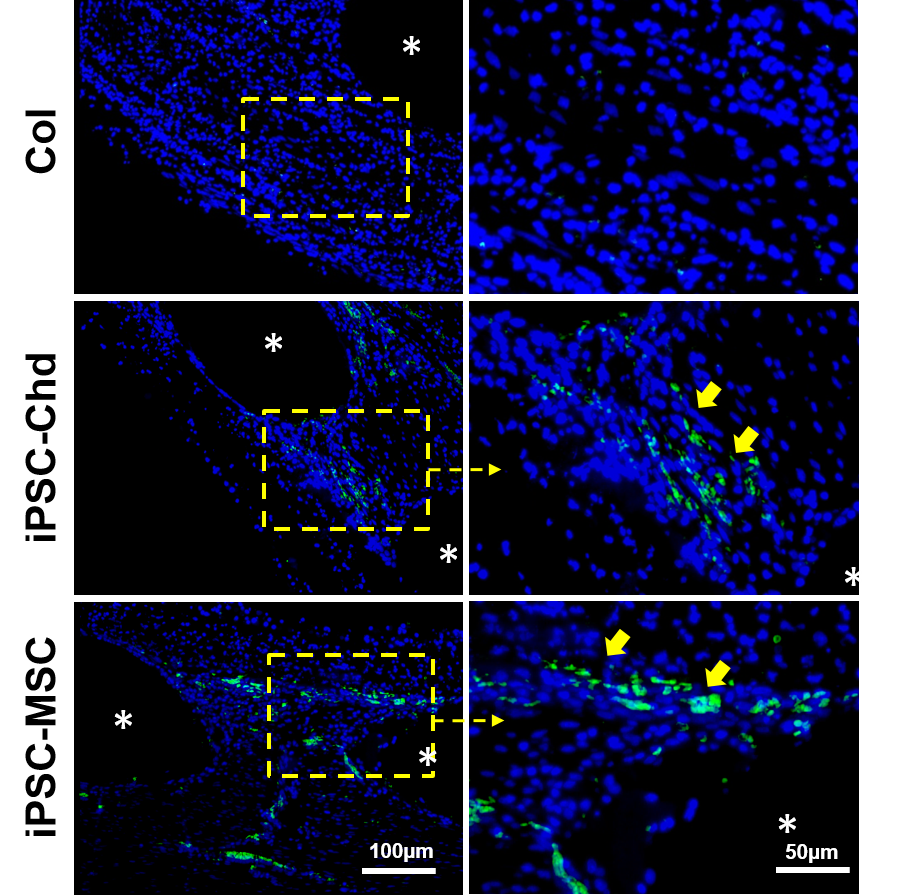


**Figure 6.** Immunohistochemistry using anti-human-nuclei antibodies for cell tracking. Human nuclei-positive cells (arrow, green) were revealed around the implanted scaffold (*, PCL strand) in all groups.


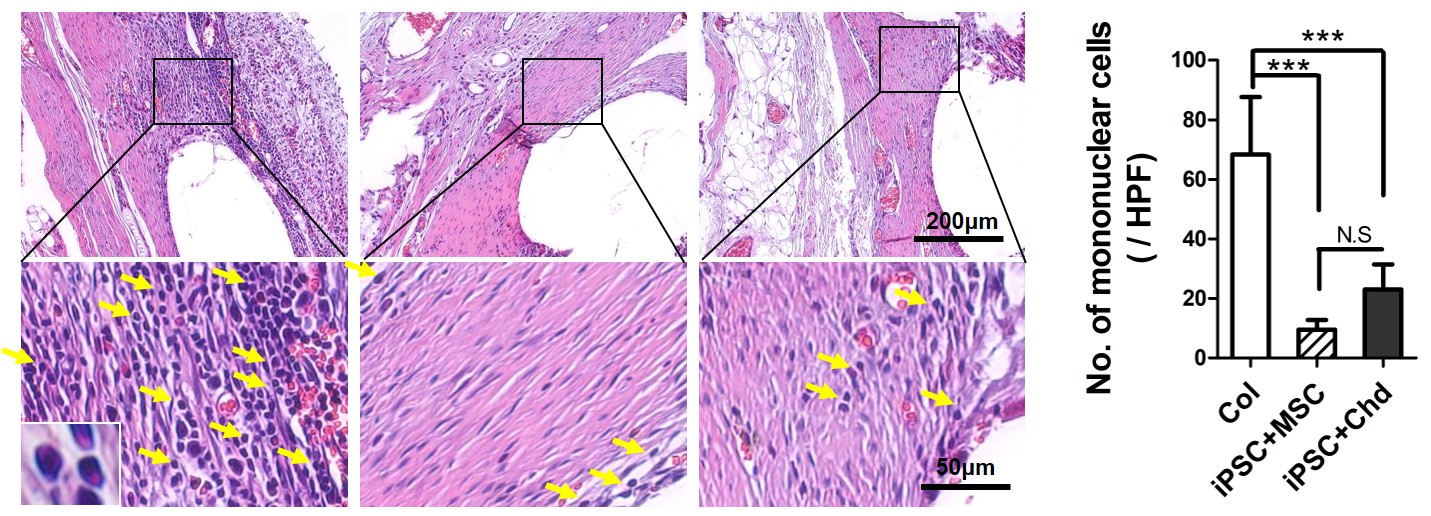


**Figure 7.** (A) Distribution of mononuclear cells via H & E staining at 28 days post-tracheal surgery. The yellow arrows indicate the mononuclear cells. (B) Statistical analysis of the number of mononuclear cells per high power field (HPF; x400)


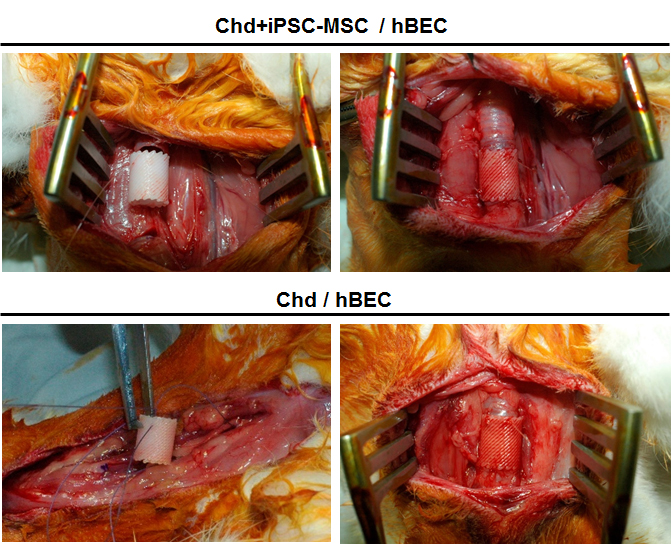


**Figure 8.** Implantation of two-layered tracheal scaffold co-seeded with chondrocytes and iPSC-MSCs into the segmental tracheal defects.


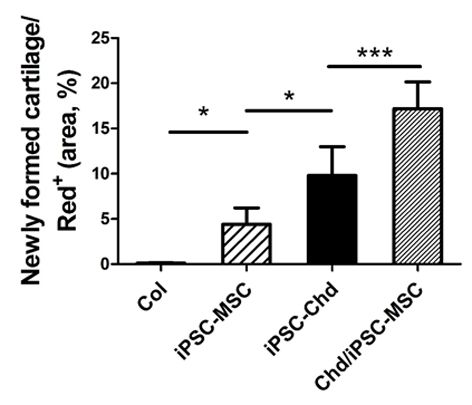


**Figure 9.** Regenerative tracheal cartilage was calculated based on the quantity of white signal within the area of the region of interest (red^+^) in micro-CT images. Newly formed cartilage was dramatically increased in the Chd/iPSC-MSC group compared with the other groups (**P* < 0.05 or ****P* < 0.001).


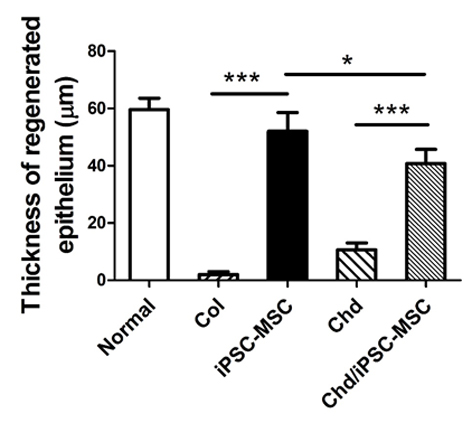


**Figure 10.** Quantitative analysis of the thickness of regenerative epithelium 4 weeks after surgery. Experimental groups seeded with iPSC-MSCs had a significantly greater epithelial thickness than the Col and Chd groups (**P* < 0.05 or ****P* < 0.001).
